# Computing of Low Shear Stress-Driven Endothelial Gene Network Involved in Early Stages of Atherosclerotic Process

**DOI:** 10.1155/2018/5359830

**Published:** 2018-09-25

**Authors:** Federico Vozzi, Jonica Campolo, Lorena Cozzi, Gianfranco Politano, Stefano Di Carlo, Michela Rial, Claudio Domenici, Oberdan Parodi

**Affiliations:** ^1^CNR Institute of Clinical Physiology, Pisa, Italy; ^2^CNR Institute of Clinical Physiology, Milan, Italy; ^3^Genetic Laboratory, Niguarda Hospital, Milan, Italy; ^4^Department of Control and Computer Engineering, Politecnico di Torino, Italy

## Abstract

**Background:**

In the pathogenesis of atherosclerosis, a central role is represented by endothelial inflammation with influx of chemokine-mediated leukocytes in the vascular wall. Aim of this study was to analyze the effect of different shear stresses on endothelial gene expression and compute gene network involved in atherosclerotic disease, in particular to homeostasis, inflammatory cell migration, and apoptotic processes.

**Methods:**

HUVECs were subjected to shear stress of 1, 5, and 10 dyne/cm^2^ in a Flow Bioreactor for 24 hours to compare gene expression modulation. Total RNA was analyzed by Affymetrix technology and the expression of two specific genes (CXCR4 and ICAM-1) was validated by RT-PCR. To highlight possible regulations between genes and as further validation, a bioinformatics analysis was performed.

**Results:**

At low shear stress (1 dyne/cm^2^) we observed the following: (a) strong upregulation of CXCR4; (b) mild upregulation of Caspase-8; (c) mild downregulation of ICAM-1; (d) marked downexpression of TNFAIP3. Bioinformatics analysis showed the presence of network composed by 59 new interactors (14 transcription factors and 45 microRNAs) appearing strongly related to shear stress.

**Conclusions:**

The significant modulation of these genes at low shear stress and their close relationships through transcription factors and microRNAs suggest that all may promote an initial inflamed endothelial cell phenotype, favoring the atherosclerotic disease.

## 1. Background

The development of atherosclerosis is commonly associated with high levels of lipids in blood able to generate the development of sclerotic plaques. New knowledge from basic and experimental science has demonstrated the role for inflammatory pathway in early events of disease pathogenesis. Different in vivo animal models of atherosclerosis show signs of inflammation, both at local than at systemic levels, associated with lipid accumulation in the artery wall. For example, leukocytes, important mediators of host defenses and inflammation, localize in earliest lesions in animal models and in humans as well [1]. The molecular mechanisms involved in lymphocytes recruitment by activated endothelial cells at atherosclerotic lesion formation sites are similar to those reported for neutrophils and monocytes [1, 2]. The different steps of atherogenic leukocyte recruitment such as rolling, adhesion, and transmigration are controlled by functionally specialized chemokines [3, 4]. Whereas soluble chemokines were first described to induce directed chemotaxis of leukocytes, chemokines trigger the integrin-mediated arrest of rolling leukocytes [5, 6]. Chemokine/chemokine receptor network is essential for direction of leukocyte migration in homeostatic and inflammatory conditions. Numerous reports described the important role of chemokines and of their receptors in the regulation of leukocyte recruitment during atherosclerosis [7]. Chemokine (C-X-C motif) Receptor 4 (CXCR4) signaling has been reported to modulate cell chemotaxis, survival [1, 8], and apoptosis [9–11]. Through this receptor the Macrophage Migration Inhibitory Factor (MIF), able to regulate inflammatory cell (T cells, monocytes) recruitment to lesion area [12, 13], expresses its proatherogenic action.

Involved in monocyte adhesion is also Intercellular Adhesion Molecule 1 (ICAM-1). Upon cytokine stimulation with Tumor Necrosis Factor alpha (TNF-alpha) [14], Angiotensin II, and Oxidized Low Density Lipoprotein (ox-LDL) [15], ICAM-1 expression increases in wall vessel cells [16, 17] producing proinflammatory effects with leukocyte recruitment.

Although the pathobiology of atherosclerosis is a complex biological multifactorial process, blood flow-induced shear stress has emerged as essential feature of atherogenesis. This fluid drag force, acting on vessel wall, is mechanotransduced into biochemical signals, resulting in vascular behavior changes. Maintenance of physiologic, laminar shear stress is known to be crucial for normal vascular functioning, which includes regulation of vascular caliber, inhibition of proliferation, thrombosis, and inflammation of the vessel wall. Nonlaminar flow promotes changes in endothelial gene expression, cytoskeletal arrangement, leukocyte adhesion, and vasoreactive, oxidative, and inflammatory states of artery [5, 18, 19]. Disturbed shear stress also influences site selectivity of atherosclerotic plaque formation and its associated vessel wall remodeling, which can affect plaque vulnerability and stent restenosis. Shear stress is critically important in regulating atheroprotective normal physiology as well as pathobiology and dysfunction of vessel wall through complex molecular mechanisms that promote atherogenesis [20].

The aim of this work is to analyze the effect of different shear stress levels in vitro on endothelial gene expression using a laminar flow bioreactor [21]. Thanks to microarray analysis, the possible involvement in early phases of atherosclerotic disease of genes significantly regulated and linked to inflammatory and apoptotic process have been observed. In particular, CXCR4, ICAM-1, Tumor Necrosis Factor Alpha-Induced Protein 3 (TNFAIP3), and Caspase-8 (CASP8) genes that present a role in atherosclerosis development at different level and with different functions were considered. With the support of bioinformatics analysis it is possible to highlight existing relationships between genes under investigation generating a potential regulation system network.

## 2. Methods

### 2.1. Endothelial Cell Culture

Primary Human Umbilical Vein Endothelial Cells (HUVECs) were isolated from human cords. Fresh human umbilical cords were recovered from healthy females at the Obstetrics and Gynecology Unit of the Azienda Ospedaliera Universitaria Pisana, after obtaining written informed consent for use of these samples in research approved by the Local Ethics Committee of Area Vasta Nord Ovest. The umbilical cords were stored in PBS at 4°C, sent to our laboratory within 1 hour of delivery, and treated anonymously conforming with the principles outlined in the Declaration of Helsinki. Umbilical vein was cannulated, washed with Phosphate Buffered Saline (PBS) solution, and filled with 3 mg/ml collagenase IV solution in PBS. After 20 min in incubator, it was washed again with Endothelial Cell Growth Medium (ECGM) (Promocell, Heidelberg, Germany) to block collagenase action and, after centrifugation (900 rpm, 5 min), pellet was recovered with fresh complete media and seeded in gelatin 1% pretreated flask for cell adhesion. Every 2 days media culture was changed, until the confluence.

### 2.2. Bioreactor

**Figure 1:**
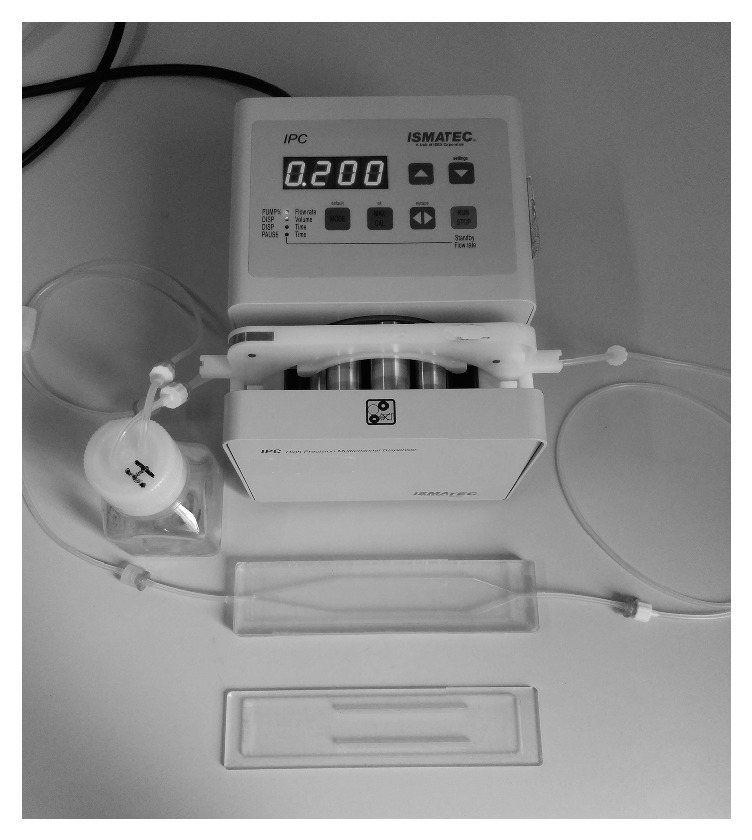
Assembled laminar flow bioreactor (LFB). Bioreactor is composed by a mixing chamber filled with a complete culture media with 5% of Dextran, a cell culture chamber, and a peristaltic pump. All the components were connected in a closed loop and put in an incubator to preserve temperature (37°C) and CO2 concentration in air (5%).

The bioreactor system [21, 22] (Figure 1) is composed by a mixing chamber, filled with 12 ml of complete culture media supplemented with 5% of Dextran (Sigma-Aldrich, St. Louis, MO, USA), a cell culture chamber, and a peristaltic pump (ISMATEC, Wertheim, Germany). All the components were connected in a closed loop and assembled system was put in incubator.

In order to obtain a central region with a well-defined and uniform wall shear stress, active cell culture chamber (70x20x2 mm^3^) shape was designed by accurate modeling analysis performed with finite element software for simulation of fluid dynamic flow [21].

The chamber is fabricated in polydimethylsiloxane (PDMS, Sylgard 184®) (Dow Corning, Midland, MI, USA), a biocompatible silicone polymer, through mill-molding.

### 2.3. Bioreactor Experimentation

For experiments, HUVECs between 2^nd^ and 5^th^ passage were used. When at confluence, cells were treated with 0.5% Trypsin (Lonza, Basel, Switzerland). Once detached from flask, endothelial cells were centrifuged at 900 rpm for 5 min. Pellet was resuspended in fresh media; cells were counted and seeded (15000 cells/cm^2^) on fibronectin 3 *μ*g/cm^2^ pretreated Thermanox slides (dimensions 2 x 6 cm^2^) (NUNC, Rochester, NY, USA).

When HUVECs covered surface slide, experiments with bioreactor started. The system was set in order to furnish different flow conditions, corresponding to shear stress values of 1, 5, and 10 dyne/cm^2^. Every experiment had duration of 24 hours; at the end, slides were recovered and cell images acquired under microscope. Then, endothelial cells were trypsinized with 200 *μ*l/slide. Once cells were detached, 1 ml of medium was added to block trypsin action and 50 *μ*l of suspension was recovered, before centrifugation, to perform CellTiter-Blue® Cell Viability Assay (Promega, Madison, USA). This assay is useful for monitoring cell viability through the ability of living cells to convert a dye (resazurin) into a fluorescent end product (resorufin, 579_ex_ /584_em_).

Finally, residual cell medium was centrifuged and the pellet was resuspended in 50 *μ*l of RNA later solution (Qiagen, Hilden, Germany) and frozen at -20°C to prevent RNA degradation until the genetic analysis.

### 2.4. Total RNA Extraction

Total RNA has been extracted from HUVECs using RNeasy® Micro Kit QIAGEN for small amounts of human cells (≤ 5 x 10^5^cells). Briefly, cell pellets were first lysed and homogenized in a highly denaturing guanidine-isothiocyanate-containing buffer and ethanol, which immediately inactivates RNases to ensure isolation of intact RNA. The lysate was passed through a RNeasyMinElute spin column, where total RNA binds to the membrane and contaminants were washed away. Traces of DNA that may copurify are removed by a DNase treatment.

The RNA quality control was performed with the Agilent BioAnalyzer system (Agilent Technologies, Santa Clara, CA, USA) that separated and subsequently detected RNA samples via laser induced fluorescence detection.

### 2.5. Microarray Analysis by Affymetrix Technology

100 ng of total RNA from each experimental set has been amplified resulting in unlabeled cDNA. An in vitro transcription reaction was performed in presence of biotin-labeled ribonucleotides mixture to produce biotinylated cRNA from the cDNA template. Biotinylated cRNA molecules were hybridized to their complementary sequences on the GeneChip surface. For each experimental condition, 2 microarrays (HG-U133-Plus 2.0, Affymetrix, Santa Clara, CA, USA) have been used; every array allows measuring the expression level of over 47,000 human transcripts, representing 38,573 gene clusters in the UniGene database plus 841 anonymous full-length transcripts, and a number of anonymous partial sequences of cDNA. The fluorescence data were processed using MicroArray Suite software version 5.0 (Affymetrix). Data from the gene microarray experiments were preprocessed using the Robust Multiarray Average (RMA) algorithms making adjustments for systematic errors introduced by differences in procedures and dye intensity by collaboration of COGENTECH (Consortium for Genomic Technologies, Milan, Italy). Microarray data have been submitted to the Gene Expression Omnibus (GEO) under accession n. GSE45225.

### 2.6. Quantitative Real-Time PCR of CXCR4 and ICAM-1

For Real-Time Polymerase Chain Reaction (RT-PCR), the reverse transcription of total RNA (500 ng) was performed using the Transcriptor First strand cDNA synthesis kit (Roche Applied Science, Indianapolis, IN, USA).

Every cDNA solution (2 *μ*l) was mixed with MgCl_2_ (3 mM), primers (0.3 *μ*M) and Hybridization Probes (0.15 *μ*M), designed ad hoc by TIBMOLBIOL (Roche), and with LightCyclerFastStart DNA Master Mix by Roche (10X). The cycling protocol consisted of denaturation step at 95°C for 10 s, annealing step at 58°C for 10 s and extension step at 72°C for 10 s. This protocol was repeated for 45 times. Fluorescence was detected at 640 nm and measured at the end of each extension step. The LightCycler software 4.05 calculated the Crossing point, (Cp*), *where the samples fluorescence curve turns sharply upward. This turning point corresponds to the first maximum of the second derivative of the curve. For final quantification of samples, an external calibration curve was obtained using specific amplicon with known concentration.

### 2.7. Preparation of Standard Curve

For absolute quantification of CXCR4 and ICAM-1 gene expressions, we prepared standard curves amplifying a fragment of CXCR4 or ICAM-1 transcript coming from a sample of cDNA with conventional PCR. The resulting fragments were purified by the QIAquick PCR Purification kit protocol (QIAgen). CXCR4 (46.1 ng/l) and ICAM-1 (25.5 ng/l) products were measured at 260 nm. Sevenfold serial dilutions of quantified ICAM-1 and CXCR4 standard amplicons were used for standard curve preparation (7 points with concentration range from 10^−4^ to 10^−10^ ng/l).

### 2.8. Statistical Analysis

After quantile normalization and filtering with a false discovery rate below 20% (FDR < 0.2), 6807 genes of 40508 analyzed were selected and clustered by Euclidean distance measure. Genes were sorted for differential expression based on one-way ANOVA and Different Expressed Genes (DEG) were identified as those genes having adjusted p values < 0.05 with Fold Change (FC) ≥ 2 fold in modulus.

### 2.9. Bioinformatics Analysis

In order to further support wet lab results and to obtain a wider understanding of the possible regulations surrounding genes in HUVECs under low shear stress, and their role in the early phases of atherosclerosis, a bioinformatics approach was performed. Regulators include both microRNA and Transcription Factors (TF) whose role is central in posttranscriptional and transcriptional regulation and whose contribution may better uncover the dynamics of the regulatory interaction that take place in the network comprising the already identified genes.

For the network enhancing process, we used ReNE (Regulatory Network Enhancer), a Cytoscape 3.x plugin we developed to automatically enrich a gene-based regulatory network by adding transcriptional, posttranscriptional, and translational data [23]. During the enhancing process, ReNE automatically retrieves regulatory information from multiple public repositories (i.e., miRIAD, TargetMine, miRanda, NCBI, UniProt, TargetHUB, PicTar, miRTarBase, TargetScan, and miRBase) and also provides a set of utilities in order to filter out the large amount of false positive predictions (usually stored in public repositories) for guaranteeing more reliable data.

## 3. Results

### 3.1. Flow Dependence of Cell Reorganization and Viability

**Figure 2:**
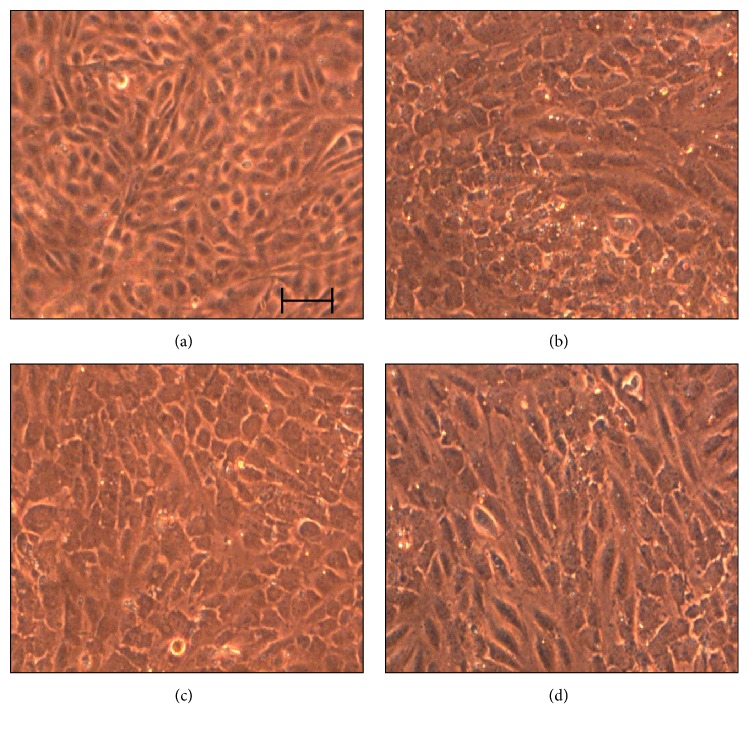
HUVECs under shear stress conditions. Picture of HUVECs subjected at 0 (a), 1 (b), 5 (c), and 10 dyne/cm^2^ (d) after 24 hours of stimulus (scale bar equal to 100 *μ*m).

Endothelial cells were photographed in order to analyze cell shape and behavior at different shear stress levels. One of the first results was the detection of flow-induced modifications on cell morphology, characterized by cytoskeletal reorganization, with prevalent cobblestone shape and initial elongated structure (Figures 2(b), 2(c), 2(d)) with respect to control (Figure 2(a)).

**Figure 3:**
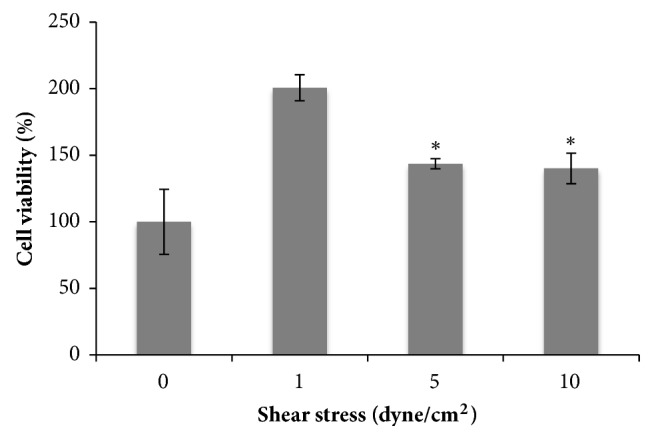
Cell viability of HUVECs after shear stress treatment. (ANOVA statistical analysis p<0,05 for: 1 versus 5; 1 versus 10).

Furthermore, cell viability presented a statistical increase in the case of 1 dyne/cm^2^ with respect to 5 and 10 dyne/cm^2^, indicating that lowest shear stress conferred proliferative behavior to endothelial cells (Figure 3).

This last result highlights a significant modulation by shear stress of cell activity, in terms of metabolic/proliferating profile but also in genetic modulation of biological mediators of our interest.

### 3.2. Microarray Results

Microarray data were published on Gene Expression Omnibus (GEO) (access number GSE45225). Globally, 3000 ID probes were found to be differentially expressed in our experimental conditions. HUVECs exposed to different shear stress values were initially compared with those that had been maintained under static conditions to verify how many gene expressions were modulated. As shown in Table 1, the used low shear stress regulated 463 ID probes with respect to static condition, while middle and high shear stress expressed 1008 and 921 different genes, respectively, compared to no flow state.

A comparison between flows may give a more accurate picture of gene expression modulation in physiological or pathological states. Low shear stress, compared to middle and high flow, regulated 142 and 397 different genes, respectively. Among 397 genes of 1 versus 10 dyne/cm^2^ stress, 206 (52%) were up- and 191 (48%) were downregulated; in 1 versus 5 dyne/cm^2^ comparison 57 (40%) were up- and 85 (60%) were downregulated.

In the comparison between low and high shear stress, we focused on genes that showed the highest up- and downregulation (selection criteria were p<0.05 and FC>2).

Microarray analysis (Table 2) highlighted a strong upregulation of CXCR4 at low (1 dyne/cm^2^) compared to high shear stress (4.53-5.54 fold variation) and significant at intermediate level (2.15-2.78 fold variation) in inversely proportional shear-dependent manner. Caspase-8 gene was significantly upregulated only at low shear stress with respect to 10 dyne/cm^2^ (2.13 fold variation). Conversely, a marked downregulation of TNFAIP3 gene (-4.19- and -3.28-fold variation) was observed. A mild reduction in gene expression (-3.33- and -3.88-fold variation) was observed also for ICAM-1 when 1 dyne/cm^2^ was compared to 10 dyne/cm^2^. In general, low shear stress seems to represent a key factor in gene expression modulation of endothelial response to fluid dynamical forces.

### 3.3. Quantitative Real-Time PCR Results

**Figure 4:**
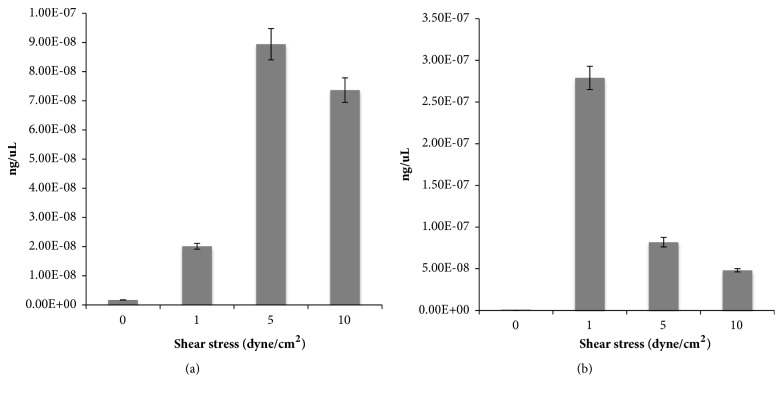
RT-PCR of ICAM-1 (a) and CXCR4 (b) genes in the different shear stress conditions.

Microarray results gave a global vision about the effects of fluid mechanical stimulation on endothelial cell culture. Focusing on the role of chemokines in early stages of atherosclerosis development, gene expressions of CXCR4 and ICAM-1 mediators were also analyzed. Results from RT-PCR confirmed the trend observed for these genes in microarray analysis. ICAM-1 mRNA levels increased from 1 to 10 dyne/cm^2^ (Figure 4(a)) while CXCR4 gene expression, high at 1 dyne/cm^2^, decreased when compared to 5 and 10 dyne/cm^2^ experiments (Figure 4(b)). Both ICAM-1 and CXCR4 mRNA concentrations are very low in static condition.

### 3.4. Bioinformatics Analysis

We used ReNE to enhance and better understand regulatory relations that take place among the previously identified genes. In order to cope with the large amount of false positive microRNA predicted targets, usually present in public repositories, we only collected microRNA targets confirmed by miRTarBase [24]. MiRTarBase collects, in fact, only experimentally validated microRNA-target interactions, which resulted in a smaller and more reliable set of microRNAs. Transcription factors have been collected from TargetMine [25].

**Figure 5:**
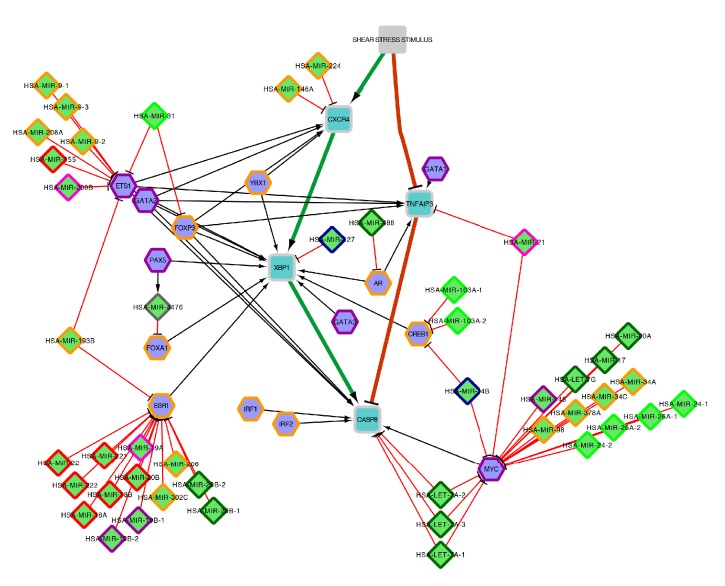
Schematic representation of the enhanced network. (i) Squares represent the genes previously identified in wet lab assessment, (ii) diamonds represent microRNAs, and (iii) hexagons represent transcription factors. The node stroke represents the role of each regulator as follows: angiogenesis (red), apoptosis (dark green), atherogenesis (light green), Inflammatory response (orange), NO synthesis and response (pink), senescence (blue), shear stress regulation (purple), and previously identified genes (light gray).

The enhanced network is depicted in Figure 5 and the list of enhanced entities (transcription factors and microRNAs) is synoptically reported in Table 3.

We validated through literature the whole set of discovered regulatory entities, to estimate both their role under shear stress conditions and the overall reliability of the resulting enhanced network. Results confirmed the expected reliability of the enhanced network, given an overall validation of 88,3% (40 out 46 microRNAs and 13 out of 14 transcription factors appear directly involved in shear stress or in shear stress induced inflammation).

Overall, the proposed enhanced network confirms the role of CXCR4, TNFAIP3, and CASP8 plus X-box Binding Protein 1 (XBP1) in shear stress, further enlarging the set of regulations and highlighting the presence of common regulatory motif composed by TFs and microRNAs, already recognized in shear stress, that possibly finely tune the pathway behavior.

## 4. Discussion

In this work, our attention was focused on first modifications occurring to endothelial cell gene profile, able to induce the development of an atherosclerotic lesion. The analysis of results shows a peculiar pattern of gene modulation linked to inflammation and we highlight a possible gene network involved in this pathological process.

The presence of low shear or nonlaminar flow is able to induce changes in gene expression profile that predispose endothelium to the initiation and development of atherosclerotic lesions [26, 27].

We decided to submit HUVECs at a well-defined range of shear stress values (0, 1, 5, and 10 dyne/cm^2^) to cover a wide spectrum of conditions, according to flow ranges employed in the majority of publications [28–31]. Physiologic fluid flows in the ranges of about 1-50 dyne/cm^2^ [32, 33] are found in vivo in the majority of blood vessels and its disturbance can produce pathogenic states such as thrombosis and atherosclerosis. It has verified that regions of arterial circulation prone to develop atherosclerosis are characterized by “disturbed,” oscillatory flow with low levels of shear stress (mean time-averaged shear stress ≤ 1 dyne/cm^2^) [34]. The range of flow we chose let us analyze physiological shear stress (5 and 10 dyne/cm^2^) with respect to a proatherogenic shear rate (1 dyne/cm^2^).

Obtained results show that shear stress modulates cell structure in terms of cytoskeletal reorganization with a cobblestone architecture at all shear stress values and with a smooth elongation at 10 dyne/cm^2^. This behavior is similar to that observed by Sakamoto [35], where only cell exposed to high shear stress showed a tendency to align parallel to the direction of flow. Our results are similar to those from literature also in terms of cell viability: a significant increase of cell metabolic activity was obtained at low shear stress, as previously observed by Conway et al. [31].

Furthermore, the comparison of different shear rates on a uniform culture of human endothelial cells by microarray analysis highlights the earliest potential modulator of the disease process.

In our genetic study, Affymetrix analysis showed a regulation of various genes, in particular linked to inflammation and apoptosis, such as CXCR4, ICAM-1, Caspase-8, and TNFAIP3 genes. These experiments highlighted a strong upregulation of CXCR4 and a >2 fold increase for Caspase-8; on the other hand, remarkable downregulation of TNFAIP3 and, less in amplitude, of ICAM-1 was evidenced. Furthermore, the expression values of two genes, CXCR4, for its highest overexpression, and ICAM-1, known to be involved in the atherosclerotic process, were confirmed with RT-PCR.

The overexpression of CXCR4 seems to indicate an active state of endothelial cells subject to a low shear stress, a first signal of “reaction” to the atheroprone waveform of flow in the bioreactor. CXCR4, as chemokine receptor, is involved in the MIF action. This is widely expressed in macrophages and endothelial cells of atherosclerotic plaques and induces integrin-independent arrest and transmigration of monocytes and T cells. MIF contributes, in this way, to lesion progression and plaque inflammation trough the interaction with CXCR4 [12, 13, 36].

The MIF involvement in atherosclerotic plaque progression was confirmed using MIF^−/−^/LDL-R^−/−^mice on an atherogenic diet for 12 and 26 weeks [37]. Interestingly, in vitro adhesion assays revealed that monocyte adhesion on ox-LDL-treated human aortic endothelial cells depends almost completely on endothelial MIF and stimulation of aortic endothelial cells with MIF for 2 h induced monocyte adhesion under flow conditions, providing preliminary hints to a chemokine-like function of MIF in leukocyte recruitment [38].

Regarding ICAM-1 gene levels, our finding shows a decrease of ICAM-1 gene expression at low shear stress, differing from previously published data [28]. These conflicting results can be explained both by the absence of an inflammatory stimuli [14, 15] and by its role in elaboration of “mature” atherosclerotic lesions [39]. On the other hand, Conway et al. [31], comparing the effects of reversing shear stress (time-average: 1 dyne/cm^2^, max: +11 dyne/cm^2^, min: -11 dyne/cm^2^, 1 Hz), arterial steady shear stress (15 dyne/cm^2^), and low steady shear stress (1 dyne/cm^2^) on surface expression of ICAM-1, cell proliferation, and monocyte adhesiveness, verified that only reversing shear stress exposure induced monocyte adhesion. ICAM-1 expression was also lower at 1 dyne/cm^2^ with respect to 15 dyne/cm^2^, confirming our results. Laminar flow produced a significant time-dependent increase in the surface expression of ICAM-1 on endothelial cells, while turbulent flow did not affect its surface expression [40, 41]. A direct link between shear stress and ICAM-1 gene expression, through the identification of a Shear Stress Response Element (SSRE) in the promoter region for the ICAM-1 gene, has been established by Resnick [42].

Considering the literature, also Caspase-8 and TNFAIP3, the other two genes most modulated in our experiments, can assume an interesting role.

Caspase-8 is an initiator of the extrinsic pathway of apoptosis. Scharner [43] demonstrated the strictly link between Caspase-8 and CXCR4. Aging with a blocker of apoptotic agent, a downregulation of CXRC4 expression on EPCs in vitro was observed. On the other hand, the role of altered shear stress, like low flow, as a regulator of cell apoptosis has recently been demonstrated by Li and Xu [44]. In endothelial cells, disturbed flow stress resulted in a sustained activation of beta-1 integrin system linked to the X-box Binding Protein 1 (XBP1), which functions as a key signal transducer resulting in increase of caspase activation in endothelial cells [45]. Referring to the literature, in our work a possible correlation between CXCR4 and Caspase-8 can be shown, furnishing an interesting new research starting point

Regarding TNFAIP3, in our experimental model, low flow downexpressed the gene, which has normally a protective function against apoptosis and inflammation through the blockade of the transcription factor, Nuclear Factor kappa-light-chain-enhancer of activated B cells (NF-kB) [46]. In cultures derived from primary cells, various investigators have found that overexpression of TNFAIP3 protects cells from cycloheximide/TNF–, ceramide/TNF–, and IL-1–mediated apoptosis [47–49]. This link between apoptosis and TNFAIP3 was also demonstrated by Daniel et al. [50]. TNFAIP3 also blunts natural killer cell-mediated EC apoptosis by inhibiting Caspase-8 activation. These data show the cytoprotective effect of TNFAIP3 in ECs. In our experiments the downregulation of TNFAIP3 may explain the correlated upregulation of Caspase-8 gene, favoring an inflammatory and proatherogenic cell condition.

The wet lab results were further exploited resorting these by bioinformatics analysis: the set of previous identified genes (i.e., CXCR4, TNFAIP3, and CASP8 plus XBP1) was enriched with the set of its upstream regulators (i.e., microRNAs and transcription factors) (Figure 5). This approach allowed deeper insight about the regulatory gene dynamics under low shear stress conditions.

The enhancing process added to the network 59 new interactors (14 transcription factors and 45 microRNAs) (Table 3), each of them has been validated in literature in order to evaluate its role and to measure the overall reliability of the enhanced network. Interestingly, all the newly introduced regulators, except one, appear strongly related to the shear stress phenomenon.

The 14 transcription factors appear evenly distributed between inflammatory response (8 out of 14: IRF2, FOXP3, YBX1, IRF1, FOXA1, CREB1, AR, and ESR1) and shear stress regulation (6 out of 14: MYC, GATA1, GATA2, GATA3, ETS1, and PAX5). In particular, ETS1, GATA2, and FOXP3 act as hubs by targeting all the previously identified genes and ESR1, MYC, and ETS1 are, in turn, coregulated by large separated groups of microRNAs each.

The 44 validated microRNAs are distributed among multiple classes reported as commonly involved in shear stress phenomenon: 13 microRNAs are involved in inflammatory response, 9 in apoptosis, 7 in both angiogenesis and atherogenesis, 3 in both NO synthesis and response and shear stress regulation, and finally, 2 in senescence.

Three large heterogeneous separated groups of microRNAs appear to act in concert to respectively regulate: ETS1 (hsa-mir-9-1, hsa-mir-9-2, hsa-mir-9-3, hsa-mir-155, hsa-mir-208a, hsa-mir-200b), ESR1 (hsa-mir-18a, hsa-mir-18b, hsa-mir-19a, hsa-mir-19b-1, hsa-mir-19b-2, hsa-mir-206, hsa-mir-20b, hsa-mir-22, hsa-mir-221, hsa-mir-222, hsa-mir-29b-1, hsa-mir-29b-2, hsa-mir-302c), and MYC (hsa-let-7g, hsa-mir-145, hsa-mir-17, hsa-mir-20a, hsa-mir-24-1, hsa-mir-24-2, hsa-mir-26a-1, hsa-mir-26a-2, hsa-mir-34a, hsa-mir-34c, hsa-mir-378a).

7 microRNAs (hsa-let-7a-1, hsa-let-7a-2, hsa-let-7a-3, hsa-mir-193b, hsa-mir-21, hsa-mir-31, hsa-mir-34b) act as regulatory bridges among highly connected regulatory clusters by regulating couple of targets each (transcription factors and genes).

The only unvalidated element is hsa-mir-4476; nevertheless such microRNA has been recently discovered and no clues about its role are actually available in literature. Giving the overall reliability of the rest of the enhanced network, the lack of validation of hsa-mir-4476 makes it a good candidate for further studies to elucidate its possible involvement in shear stress.

Furthermore, hsa-mir-4476 is coexpressed by PAX5 and appears participating in incoherent feed-forward loop regulatory motif [51]. Such motif acts as a pulse generator and response accelerator: PAX5 activates XBP1, but also represses XBP1 by the repression of FOXA1 mediated by hsa-mir-4476. As a result, when a signal causes PAX5 to assume its active conformation, XBP1 is rapidly produced. However, after some time, hsa-mir-4476 levels accumulate to reach the repression threshold for the XBP1 promoter. As a result, XBP1 production decreases and its concentration drops, resulting in pulse-like dynamics.

**Figure 6:**
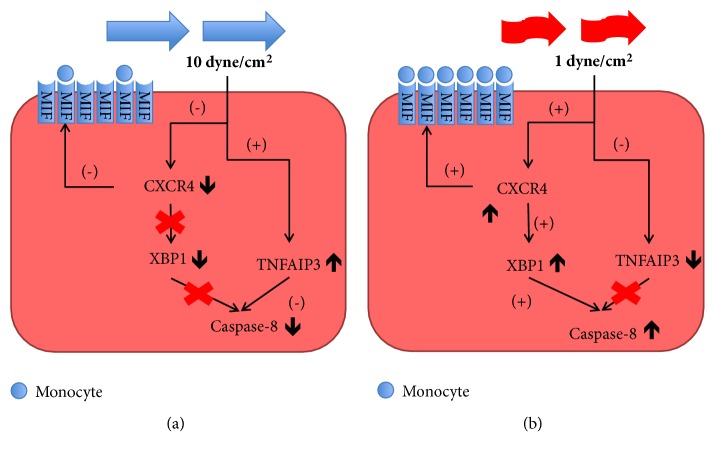
Representation of relations between CXCR4, TNFAIP3, and Caspase-8 in HUVECs under physiological and low shear stress. At physiological level (a), shear stress is able to downregulate CXCR4, limiting its role in XBP-1 stimulation with reduction of apoptotic activity of Caspase-8; in parallel, there is low interaction with MIF, reducing monocyte adhesion and trans-migration in vascular wall. TNFAIP3 is highly expressed and inhibits Caspase-8. This behavior changes when endothelial cells are subjected to low shear stress (b): CXCR4 is overexpressed, promoting its interaction with MIF and consequent increased monocyte adhesion. Furthermore, it stimulates XBP-1, with augmented apoptotic activity not counteracted by TNFAIP3, which is downregulated.

Integrating our data with the state of the art related to genes under investigation, a potential relation between CXCR4, Caspase-8, and TNFAIP3 in the development of the early phases of atherosclerotic disease can be shown (Figure 6).

In fact, endothelial chemokine receptor CXCR4 activation at low shear stress (Figure 6(b)) can play a role in the development of atherosclerosis acting as an important mediator of the endothelial response to damage through interaction with MIF.

The concomitant overexpression of Caspase-8, involved in early phase of apoptotic process, seems closely related to upregulation of CXCR4 and the downregulation of TNFAIP3 suggest that all these gene modulations can synergistically promote an initial inflamed cell phenotype at low flow with a cascade of events that come ahead the endothelial activation through adhesion molecules expression.

ICAM-1 downregulation, instead, can be justified by its limited role in the very early stages of disease, which become more important in following phases of atherosclerosis [50, 52].

## 5. Conclusions

Atherosclerotic process is a pathological event where several inputs participate to create a network with several players. In particular, our findings may shed light on specific patterns: the role of specific mediators of chemokine, caspase, and inflammatory systems in situations of low shear stress that might be relevant in the initial phases of atherosclerotic process and for fundamental regulatory action of laminar shear stress in preservation of endothelial cell integrity and survival.

Although our work presents some limitations, like RT-PCR gene analysis focused only on CXCR4 and ICAM-1 and no experiments focused on the possible cause-effect relationship of this hypothesis, it highlights that the concomitant modulation of CXCR4, Caspase-8, ICAM-1, and TNFAIP3 at low shear stress may predispose endothelium to a preinflamed phase.

At the same time, the emerging findings together with an integrated approach by the way of bioinformatics tools, which has enriched the network of specific genes, transcription factors, and microRNAs, improve knowledge about comprehension of chemokine role in early stages of patient plaque development focalizing a chemokine hypothesis-driven to be analyzed with further experiments.

## Figures and Tables

**Table 1:** Number of differentially expressed genes between conditions.

**Comparison**	**Factor considered**	**Selection criterion**	**Number of DEG**
0 vs 1	Flow effect	p<0.05 & |FC|>2	463
0 vs 5	Flow effect	p<0.05 & |FC|>2	1008
0 vs 10	Flow effect	p<0.05 & |FC|>2	921
1 vs 5	Flow effect	p<0.05 & |FC|>2	142
1 vs 10	Flow effect	p<0.05 & |FC|>2	397

**Table 2:** Fold variations of genes under investigation with microarray technique.

**Probeset ID**	**Gene**	**Fold variation 1 vs 5**	**Fold variation 1 vs 10**
209201_x_at	CXCR4	2.76	5.42
217028_at	CXCR4	2.78	5.54
211919_s_at	CXCR4	2.15	4.53
213373_s_at	CASP-8	-	2.13
202643_s_at	TNFAIP3	-2.09	-3.28
202644_s_at	TNFAIP3	-2.71	-4.19
202637_s_at	ICAM-1	-2.10	-3.88
202638_s_at	ICAM-1	-2.05	-3.33

**Table 3:** List of microRNA and transcription factors added to the regulatory network after the enhancing process. Node represents the regulator's name, type describes if the regulator is a transcription factor or a microRNA, PMID is the PubMed ID of the reference paper, and Function contains the overall regulatory function.

**Node**	**Type**	**PMID**	**Function**
HSA-MIR-155	External Mirna	23512606	Angiogenesis
HSA-MIR-18A	External Mirna	21171923	Angiogenesis
HSA-MIR-18B	External Mirna	21171923	Angiogenesis
HSA-MIR-20B	External Mirna	24158362	Angiogenesis
HSA-MIR-22	External Mirna	23512606	Angiogenesis
HSA-MIR-221	External Mirna	23512606	Angiogenesis
HSA-MIR-222	External Mirna	23512606	Angiogenesis
HSA-LET-7A-1	External Mirna	21171923	Apoptosis
HSA-LET-7A-2	External Mirna	21171923	Apoptosis
HSA-LET-7A-3	External Mirna	21171923	Apoptosis
HSA-LET-7G	External Mirna	21171923	Apoptosis
HSA-MIR-17	External Mirna	21171923	Apoptosis
HSA-MIR-20A	External Mirna	21171923	Apoptosis
HSA-MIR-29B-1	External Mirna	21171923	Apoptosis
HSA-MIR-29B-2	External Mirna	21171923	Apoptosis
HSA-MIR-488	External Mirna	21710544	Apoptosis
HSA-MIR-103A-1	External Mirna	22267480	Atherogenesis
HSA-MIR-103A-2	External Mirna	22267480	Atherogenesis
HSA-MIR-24-1	External Mirna	23729638	Atherogenesis
HSA-MIR-24-2	External Mirna	23729638	Atherogenesis
HSA-MIR-26A-1	External Mirna	22267480	Atherogenesis
HSA-MIR-26A-2	External Mirna	22267480	Atherogenesis
HSA-MIR-31	External Mirna	23729638	Atherogenesis
HSA-MIR-146A	External Mirna	21171923	Inflammatory response
HSA-MIR-193B	External Mirna	23729638	Inflammatory response
HSA-MIR-206	External Mirna	23381794	Inflammatory response
HSA-MIR-208A	External Mirna	23797034	Inflammatory response
HSA-MIR-224	External Mirna	22178270	Inflammatory response
HSA-MIR-302C	External Mirna	21490602	Inflammatory response
HSA-MIR-34A	External Mirna	21171923	Inflammatory response
HSA-MIR-34C	External Mirna	26779287	Inflammatory response
HSA-MIR-378A	External Mirna	25104350	Inflammatory response
HSA-MIR-9-1	External Mirna	23797034	Inflammatory response
HSA-MIR-9-2	External Mirna	23797034	Inflammatory response
HSA-MIR-9-3	External Mirna	23797034	Inflammatory response
HSA-MIR-98	External Mirna	25623956	Inflammatory response
HSA-MIR-19A	External Mirna	22047531	NO synthesis and response
HSA-MIR-200B	External Mirna	23975169	NO synthesis and response
HSA-MIR-21	External Mirna	22047531	NO synthesis and response
HSA-MIR-127	External Mirna	24282530	Senescence
HSA-MIR-34B	External Mirna	20424141	Senescence
HSA-MIR-145	External Mirna	23512606	Shear stress regulation
HSA-MIR-19B-1	External Mirna	25012134	Shear stress regulation
HSA-MIR-19B-2	External Mirna	25012134	Shear stress regulation
AR	Transcription Factor	26097556	Inflammatory response
CREB1	Transcription Factor	25883219	Inflammatory response
ESR1	Transcription Factor	26808832	Inflammatory response
FOXA1	Transcription Factor	24801505	Inflammatory response
FOXP3	Transcription Factor	21873519	Inflammatory response
IRF1	Transcription Factor	23934855	Inflammatory response
IRF2	Transcription Factor	2475256	Inflammatory response
YBX1	Transcription Factor	23872051	Inflammatory response
ETS1	Transcription Factor	18636553	Shear stress regulation
GATA1	Transcription Factor	15231498	Shear stress regulation
GATA2	Transcription Factor	15231498	Shear stress regulation
GATA3	Transcription Factor	15231498	Shear stress regulation
MYC	Transcription Factor	9598824	Shear stress regulation
PAX5	Transcription Factor	19616560	Shear stress regulation

## Data Availability

The microarray dataset supporting the conclusions of this article is available in the NCBI-GEO repository http://www.ncbi.nlm.nih.gov/geo/query/acc.cgi?acc =GSE45225.

## Abbreviations

CASP8: Caspase-8
Cp: Crossing point
CXCR4: Chemokine (C-X-C motif) Receptor 4
DEG: Different Expressed Genes
ECGM: Endothelial Cell Growth Medium
FC: Fold Change
GEO: Gene Expression Omnibus
HUVECs: Human Umbilical Vein Endothelial Cells
ICAM-1: Intercellular Adhesion Molecule 1
MIF: Macrophage Migration Inhibitory Factor
NF-kB: Nuclear Factor kappa-Light-Chain-Enhancer of Activated B Cells
Ox-LDL: Oxidized Low Density Lipoprotein
PBS: Phosphate Buffered Saline
PDMS: Polydimethylsiloxane
ReNE: Regulatory Network Enhancer
RMA: Robust Multiarray Average
RT-PCR: Real-Time Polymerase Chain Reaction
TF: Transcription Factors
TNF-alpha: Tumor Necrosis Factor alpha
TNFAIP3: Tumor Necrosis Factor Alpha-Induced Protein 3
XBP-1: X-box Binding Protein 1.
